# R‐spondin‐3 is an oncogenic driver of poorly differentiated invasive breast cancer

**DOI:** 10.1002/path.5999

**Published:** 2022-09-15

**Authors:** Eline J ter Steege, Mandy Boer, Nikki C Timmer, Carola ME Ammerlaan, Ji‐Ying Song, Patrick WB Derksen, John Hilkens, Elvira RM Bakker

**Affiliations:** ^1^ Department of Pathology University Medical Center Utrecht Utrecht The Netherlands; ^2^ Department of Molecular Genetics The Netherlands Cancer Institute Amsterdam The Netherlands; ^3^ Department of Experimental Animal Pathology The Netherlands Cancer Institute Amsterdam The Netherlands

**Keywords:** R‐spondin, RSPO3, breast cancer, mouse mammary gland, Wnt1

## Abstract

R‐spondins (RSPOs) are influential signaling molecules that promote the Wnt/β‐catenin pathway and self‐renewal of stem cells. Currently, RSPOs are emerging as clinically relevant oncogenes, being linked to cancer development in multiple organs. Although this has instigated the rapid development and testing of therapeutic antibodies targeting RSPOs, functional evidence that RSPO causally drives cancer has focused primarily on the intestinal tract. Here, we assess the oncogenic capacity of RSPO in breast cancer in a direct fashion by generating and characterizing a novel mouse model with conditional *Rspo3* expression in the mammary gland. We also address the prevalence of *RSPO* gene alterations in breast cancer patients. We found that a quarter of breast cancer patients harbor *RSPO2*/*RSPO3* copy number amplifications, which are associated with lack of steroid hormone receptor expression and reduced patient survival. Foremost, we demonstrate the causal oncogenic capacity of RSPO3 in the breast, as conditional *Rspo3* overexpression consistently drives the development of mammary adenocarcinomas in our novel *Rspo3* breast cancer model. RSPO3‐driven mammary tumors typically show poor differentiation, areas of epithelial‐to‐mesenchymal transition, and metastatic potential. Given the reported interplay in the Wnt/β‐catenin pathway, we comparatively analyzed RSPO3‐driven mouse mammary tumors versus classical WNT1‐driven analogues. This revealed that RSPO3‐driven tumors are distinct, as the poorly differentiated tumor morphology and metastatic potential were observed in RSPO3‐driven tumorigenesis exclusively, further substantiated by differentiating gene expression profiles. Co‐expression of *Rspo3* and *Wnt1* transduced mammary tumors with a mixed phenotype harboring morphological features characteristic of both transgenes. In summary, we report that a quarter of breast cancer patients harbor *RSPO2/RSPO3* copy number gains, and these patients have a worse prognosis, whilst providing *in vivo* evidence that RSPO3 drives poorly differentiated invasive breast cancer in mice. Herewith, we establish RSPO3 as a driver of breast cancer with clinical relevance, proposing RSPO3 as a novel candidate target for therapy in breast cancer. © 2022 The Authors. *The Journal of Pathology* published by John Wiley & Sons Ltd on behalf of The Pathological Society of Great Britain and Ireland.

## Introduction

R‐spondin proteins (RSPO1–4) are secreted ligands that have emerged as multipotent signaling molecules. Among their activities, potentiation of the Wnt/β‐catenin pathway in cooperation with Wnt ligands has been established best. As RSPO ligands act through the LGR4, LGR5, and LGR6 transmembrane receptors typically expressed on stem and progenitor cells, they play an influential role by potentiating Wnt/β‐catenin signaling and proliferation in various stem cell compartments [1, 2]. Despite the fact that RSPOs cooperate with Wnt ligands to drive canonical Wnt/β‐catenin signaling, RSPO and Wnt ligands also exert distinct, non‐interchangeable roles in the intestinal stem cell niche [3]. Here, RSPOs actively fuel self‐renewal and expansion of stem cells, dictating the size of the stem cell pool, in contrast to Wnt ligands that are unable to induce stem cell self‐renewal [3]. In line with the instrumental role of RSPOs in stem cell regulation, aberrant RSPO activation has been increasingly implicated in carcinogenesis over the last decade [4]. The oncogenic role of RSPO has been especially recognized for the intestinal tract, as mouse studies have provided functional evidence that aberrant *Rspo* expression causally drives intestinal tumorigenesis, associated with aberrant expansion of the proliferative stem cell compartment [5, 6]. Moreover, a gain in *RSPO2* or *RSPO3* levels is evident in a subpopulation of colorectal cancer patients, caused either by stromal overexpression or by specific gene fusions, among which *EIF3E–RSPO2* and *PTPRK–RSPO3* occur mutually exclusively with classical *APC* and *CTNNB1* driver mutations [7, 8, 9, 10, 11, 12, 13]. These findings put forward RSPO2 and RSPO3 as novel, clinically relevant cancer drivers in the intestinal tract, which has accordingly been recognized by a clinical trial targeting RSPO3 in colorectal cancer [14].

As RSPOs have been implicated in many cancer types, the potential clinical utility extends beyond the intestinal tract, therefore urging further investigation. Among these other types is breast cancer, which represents a different cancer type in which the steroid hormone receptors ER and PR play a crucial role in stratifying therapeutic treatment options, reflecting the instrumental role of hormonal regulation in the mammary epithelium. Hence, in the normal mammary gland, upstream steroid hormone signals are instructive in regulating mammary stem cell dynamics, and Wnt/β‐catenin signaling also plays an important stem cell regulatory role [4, 15, 16, 17]. Importantly, mouse studies have indicated that the dictating role of upstream steroid hormone signals is executed through a collaborative Rspo1–Wnt4 signaling axis that potentiates Wnt/β‐catenin signaling and stimulates self‐renewal of mammary stem cells in the normal mammary gland [18, 19]. Wnt/β‐catenin signaling also contributes to mouse mammary cancer, initially found through the identification of *Wnt1* as a mammary oncogene [20]. Transgenic mouse studies have shown that hyperactivation of the Wnt/β‐catenin pathway, e.g. in MMTV‐*Wnt1* and MMTV‐ΔN89‐β‐catenin mice, causes the development of mammary tumors [21, 22]. In breast cancer patients, especially of triple‐negative subtype, overactivation of the Wnt/β‐catenin pathway has been frequently reported; however, the underlying mechanisms responsible remain obscure as *APC* and *CTNNB1* mutations are rarely found [4, 23, 24, 25]. Wnt pathway activation may be rather achieved by alterations in alternative pathway members, as reported for Wnt antagonists [26, 27, 28, 29]. Alterations in RSPOs might present another explanation. Overexpression of *RSPO2*, *RSPO3*, and *RSPO4* has been reported in breast cancer patients, in particular in triple‐negative tumors, where enhanced *RSPO2* expression was associated with reduced metastasis‐free survival [30, 31]. *RSPO* fusions were not detected in 446 breast tumors screened by Coussy *et al* [30]. However, the triple‐negative breast cancer cell line BT549 harbors the *EIF3E–RSPO2* gene fusion. From earlier MMTV insertional mutagenesis screens in mice, *Rspo1*, *Rspo2*, and *Rspo3* had already been proposed as potential mammary oncogenes [32, 33, 34, 35]. Despite these data suggesting a pro‐tumorigenic role for RSPOs in breast cancer, functional *in vivo* evidence for their causal oncogenic capacity has remained limited. In this regard, we exploited a validated transgenic *Rspo3* mouse model to investigate RSPO3 as a paradigm. We demonstrate that a gain in RSPO3 causes the development of poorly differentiated invasive mammary tumors in mice, providing functional evidence for the causal oncogenic capacity of RSPO3 in driving breast cancer. We also show that mammary tumors driven by RSPO3 are morphologically and molecularly distinct from WNT1‐driven tumors, with higher metastatic potential. These findings suggest that RSPO3 potentially represents a novel candidate therapy target for breast cancer patients with a gain in *RSPO3*.

## Materials and methods

### *In silico* copy number analysis

Copy number analysis was performed using the METABRIC breast cancer patient dataset and the cBioPortal for Cancer Genomics (http://cbioportal.org).

### Mouse strains and tumor study

We generated the *Rspo3* ^inv^ mouse model on a 129/Ola background previously [official 129P2‐Gt(Rosa)26Sor^tm6(CAG‐Rspo3)Nki^/A (MGI:5697338, abbreviated to *Rspo3* ^inv^)], of which a detailed description is provided in ref 5. In the *Rspo3* ^inv^ mouse line, the *Rspo3* coding sequence is present in the antisense orientation between two sets of non‐homologous *Lox* sites in a head‐to‐head orientation (Figure 2A and supplementary material, Figure S1A). In the current study, *Rspo3* ^inv^ mice (129/Ola) were crossbred with MMTV‐*Cre*;MMTV‐*Wnt1* mice [22, 36] (FVB), generating required cohorts on an F1 hybrid background (maintaining all alleles heterozygous). The cohorts comprised single transgenic *Rspo3* ^inv^ control females (no transgenic expression), double transgenic MMTV‐*Cre*;*Rspo3* ^inv^ (transgenic *Rspo3* expression) and MMTV‐*Wnt1*;*Rspo3* ^inv^ (transgenic *Wnt1* expression) females, and triple transgenic MMTV‐*Cre*;*Rspo3* ^inv^;MMTV‐*Wnt1* females (transgenic *Rspo3/Wnt1* co‐expression). Mice of all genotypes were forced bred and monitored for tumor development up to a maximum age of 600 days. All animal experiments were performed according to Dutch legislation and with approval of the Animals Ethics Committee (DEC08.061).

### Histology and immunohistochemistry

Tissues were fixed in formalin or EAF (ethanol, acetic acid, and formalin mixture) and paraffin‐embedded, followed by hematoxylin and eosin (H&E) staining according to routine protocols. For the postmortem analysis of lung metastases, paraffin‐embedded lungs were sectioned and H&E‐stained at five different levels throughout the lungs. Immunohistochemistry was performed using rabbit anti‐cytokeratin‐5 (1:500, PRB‐160P; Covance, Princeton, NJ, USA), rat anti‐cytokeratin‐8 [1:1500, Troma‐I; Developmental Studies Hybridoma Bank (DSHB), Iowa City, IA, USA], rabbit anti‐ERα (1:1000, sc‐542; Santa Cruz Biotechnology, Dallas, TX, USA), and rabbit anti‐PR (1:300, RM‐9102; Thermo Fisher Scientific, Waltham, MA, USA).

### RNA isolation, cDNA synthesis, and expression analysis of whole tissue

For RT‐PCR analysis, RNA was isolated from mammary tissues using a TissueLyser LT (Qiagen, Hilden, Germany) and an RNeasy Plus Mini Kit (Qiagen), and cDNA was generated using the Maxima First Strand cDNA Synthesis Kit (Thermo Fisher Scientific). RT‐PCR was performed with MyTaq Red DNA Polymerase (GC biotech, Waddinxveen, The Netherlands) using the following primer sequences: Sense *Rspo3* F 5′ TGGGCAACGTGCTGGTTATT 3', Sense *Rspo3* R 5′ CCTATCTGCTTCATGCCAATCC 3', *Actb* F 5′ TGAGACCTTCAACACCCCAG 3', *Actb* R 5′ GAGCCAGAGCAGTAATCTCC 3'. RNA sequencing of mouse mammary tumor tissues was performed using Illumina HiSeq2000 platforms (Illumina, San Diego, CA, USA) as previously described [5]. Limma's Voom was used for normalization and normalized expression values were statistically analyzed using the Benjamini–Hochberg method in R [5]. Gene ontology analysis was performed using Qiagen Ingenuity Pathway Analysis.

## Results

### *RSPO2* and *RSPO3* copy number amplifications are associated with poor breast cancer prognosis

**Figure 1:**
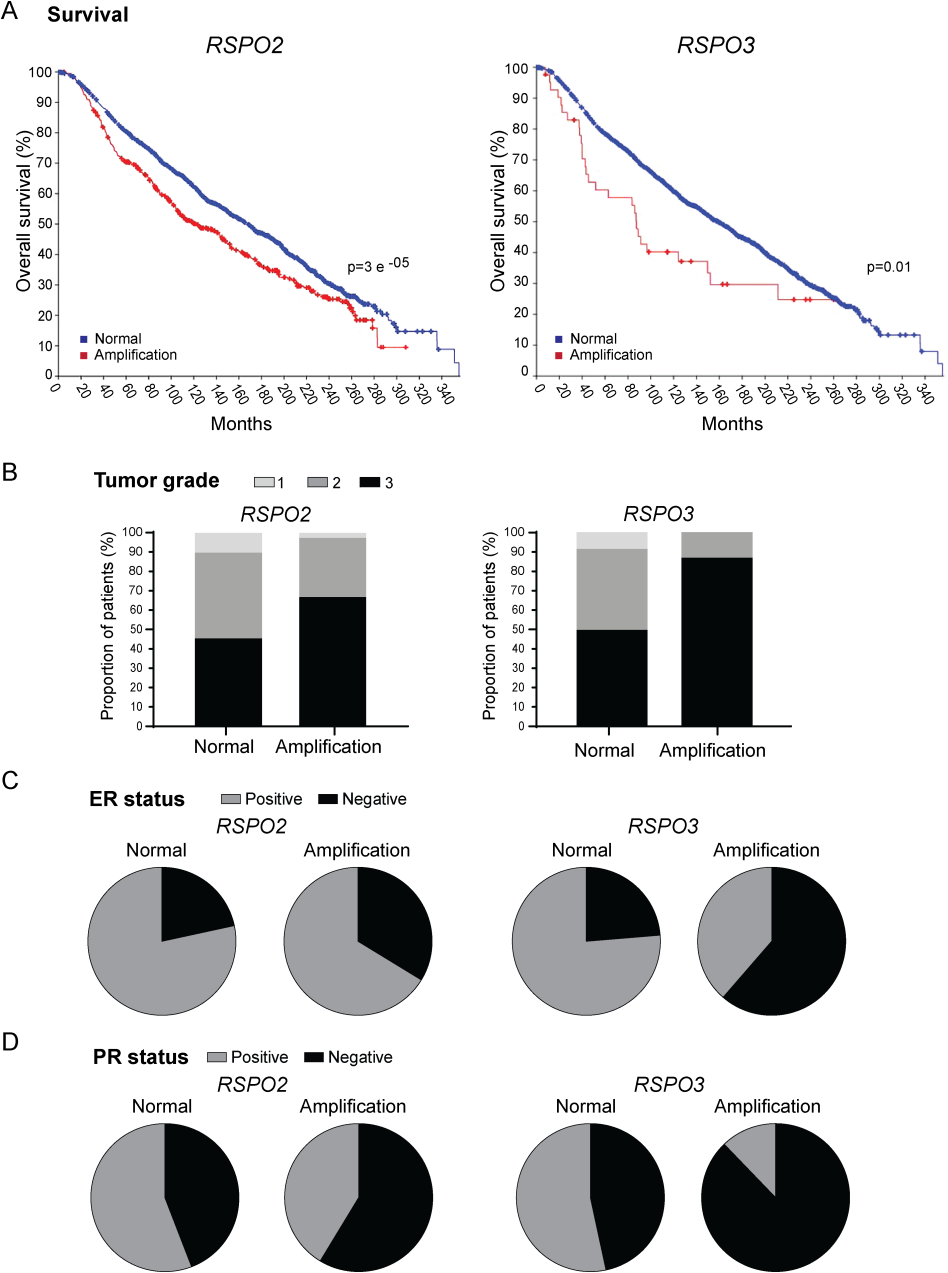
*RSPO2* and *RSPO3* copy number amplification in breast cancer patients. Copy number amplification of *RSPO2* and *RSPO3* is associated with (A) reduced overall survival (log‐rank test), (B) histological tumor grade, and lack of (C) ER and (D) PR expression.

We analyzed all four *RSPO* genes for the occurrence of copy number alterations in the METABRIC breast cancer dataset. Among the *RSPO* members, copy number amplifications of the *RSPO2* gene occurred most frequently, being present in 23% (503/2,173) of breast cancer patients. In addition, 1% harbored copy number amplifications of *RSPO1* (26/2,173), 2% of *RSPO3* (47/2,173), and 2% of *RSPO4* (48/2,173). Importantly, breast cancer patients harboring *RSPO2* or *RSPO3* copy number amplification showed a significantly reduced overall survival (Figure 1A). In line with these results, the presence of *RSPO2* and *RSPO3* copy number amplifications was associated with higher histological tumor grade (Figure 1B) and lack of expression of the steroid hormone receptors ER (Figure 1C) and PR (Figure 1D). Taken together, these data indicate that over a  quarter of breast cancer patients harbor *RSPO2* or *RSPO3* amplification, leading to a reduced clinical outcome.

### Conditional *Rspo3* expression drives mammary tumorigenesis

**Figure 2:**
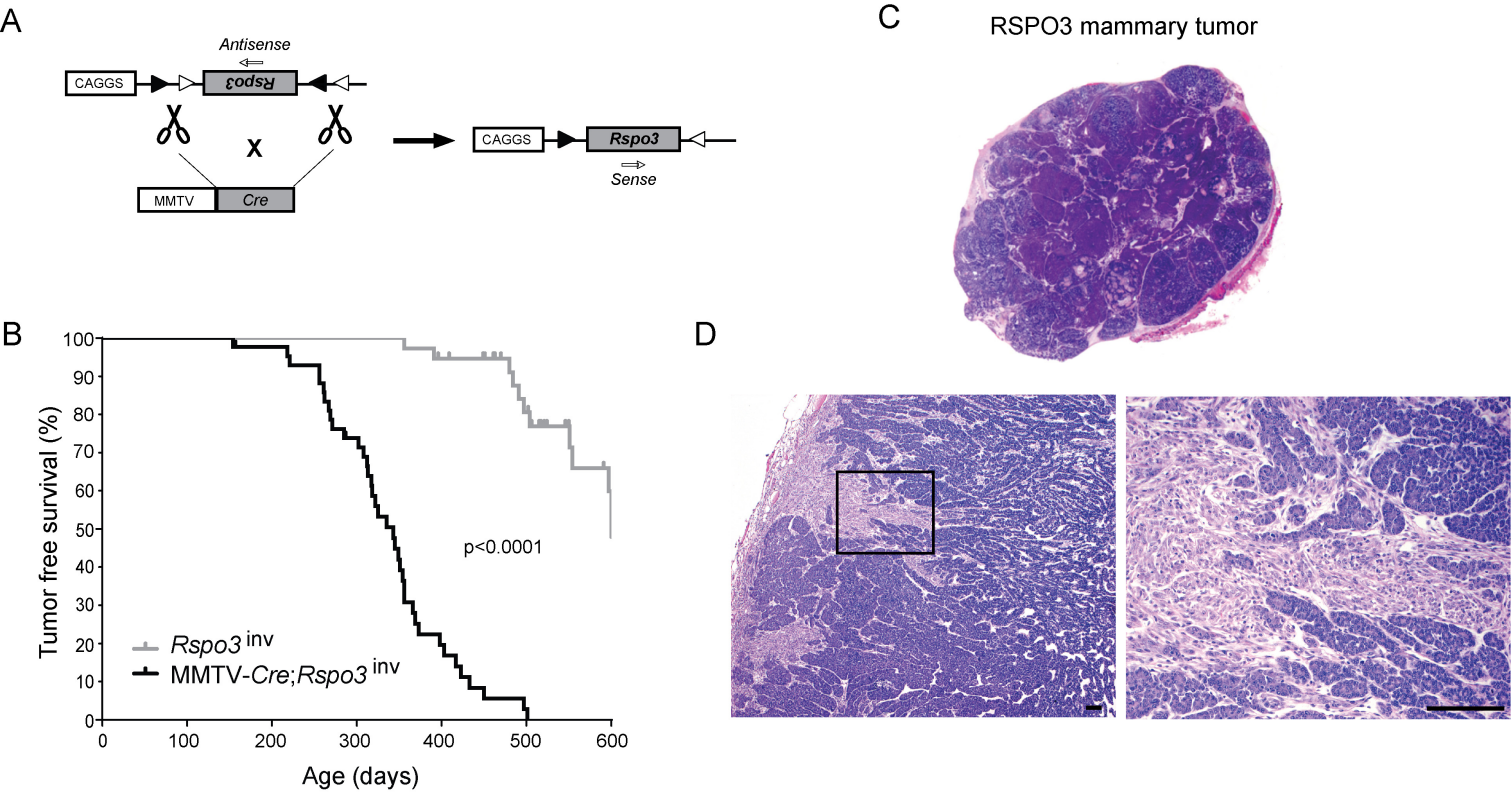
Conditional *Rspo3* mouse model of breast cancer. (A) Schematic representation of the *Rspo3* ^inv^ mouse model in which the *Rspo3* transgene is present in the antisense orientation between two pairs of *Lox* sites. Crossbreeding with MMTV‐*Cre* allows Cre‐mediated inversion of the *Rspo3* transgene into the sense orientation in the mammary gland. Adapted from ref 5 with permission of BMJ Publishing Group Ltd. (B) Survival curves of double transgenic MMTV‐*Cre*;*Rspo3* ^inv^ mice (*n* = 43) versus single transgenic *Rspo3* ^inv^ control mice (*n* = 42) (log‐rank test). (C) Scan of total and (D) microscopic pictures (4× and 20× objective) of H&E‐stained mammary tumors of MMTV‐*Cre*;*Rspo3* ^inv^ female mice.

As patient data suggested a pro‐tumorigenic role for RSPO2 and RSPO3 in breast cancer, we aimed to determine the oncogenic potential of RSPO in breast cancer. For this purpose, we used the conditional *Rspo3* ^inv^ mouse model that we generated and validated previously [5]. In this transgenic mouse model, the *Rspo3* coding sequence is placed in the inverse orientation between two sets of *Lox* sites, preventing transgene expression in this antisense configuration (Figure 2A and supplementary material, Figure S1A). By providing directed Cre recombinase activity, the *Rspo3* transgene is inverted into the sense orientation, leading to overexpression. To investigate the consequences of *Rspo3* overexpression in the mammary gland, this *Rspo3* ^inv^ mouse model was combined with MMTV‐*Cre* mice [36], providing abundant Cre expression throughout the mammary epithelium. Efficient conditional expression of transgenic *Rspo3* was confirmed in mammary gland tissues of double transgenic MMTV‐*Cre*;*Rspo3* ^inv^ mice, whereas single transgenic *Rspo3* ^inv^ control mice did not express the *Rspo3* transgene (supplementary material, Figure S1B), demonstrating its correct regulation.

To assess the oncogenic capacity of RSPO3 in the mammary gland, we generated a cohort of MMTV‐*Cre*;*Rspo3* ^inv^ double transgenic females (*n* = 43) and a corresponding control cohort of single transgenic *Rspo3* ^inv^ females (*n* = 42). MMTV‐*Cre*;*Rspo3* ^inv^ female mice developed mammary tumors consistently, providing *in vivo* evidence for the causal oncogenic capacity of RSPO3 in the mammary gland. Accordingly, the tumor‐free survival of MMTV‐*Cre*;*Rspo3* ^inv^ females was reduced to a median of 343 days, compared with 600 days in the control cohort that lacked transgenic expression (Figure 2B). The mammary tumors that developed in MMTV‐*Cre*;*Rspo3* ^inv^ mice (i.e. RSPO3‐driven tumors) macroscopically appeared as solid, compact structures, confirmed microscopically by H&E staining (Figure 2C,D). RSPO3‐driven mammary tumors typically presented as adenocarcinomas with mixed solid acinar and ductal arrangements, focal regions of squamous metaplasia, and areas with epithelial‐to‐mesenchymal transition (EMT) (Figure 2D).

### RSPO3‐driven murine breast tumors are poorly differentiated and invasive

**Figure 3:**
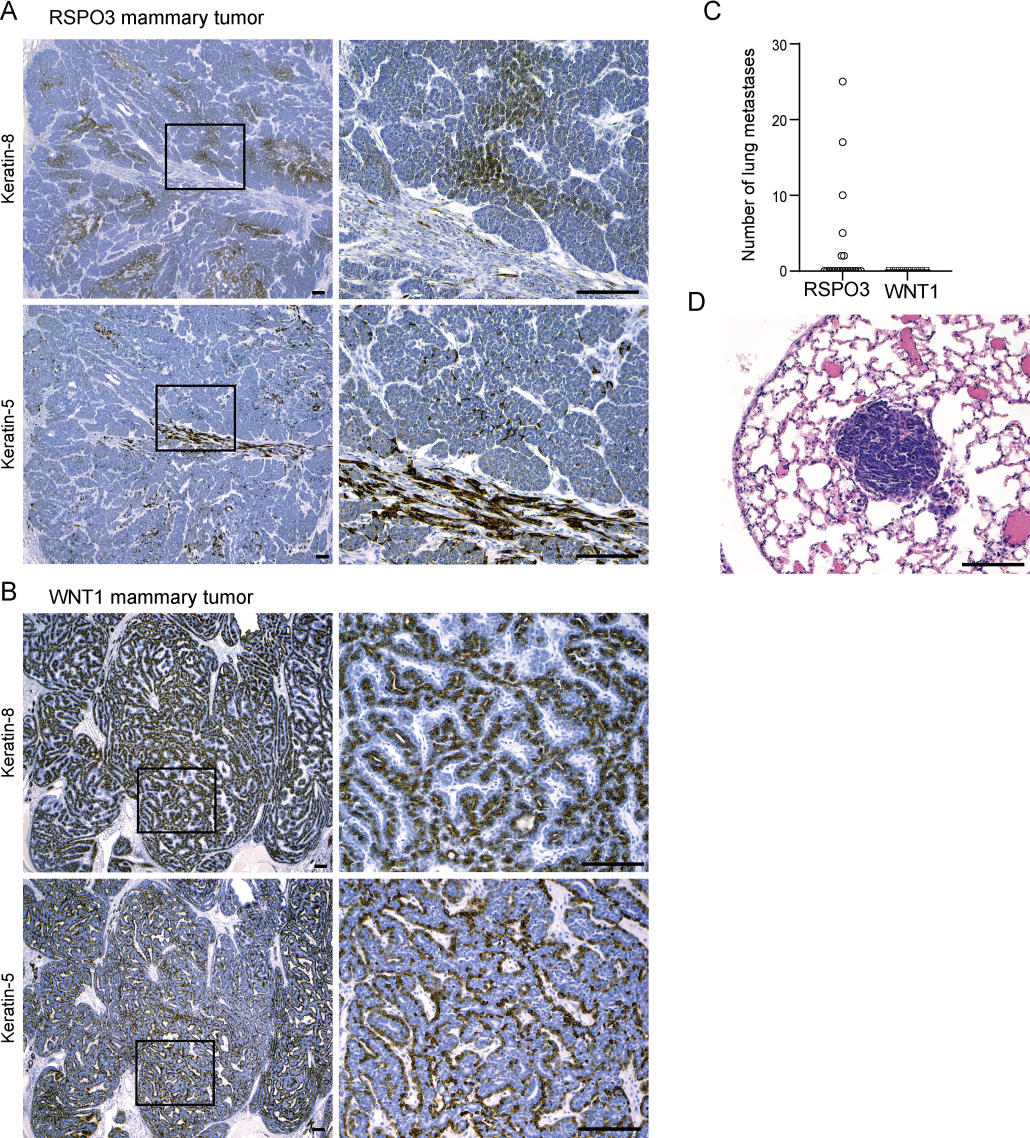
RSPO3‐driven mammary tumors are less differentiated and more metastatic than WNT1‐driven tumors. Immunohistochemical staining for K8 and K5 in mammary tumors developing in (A) MMTV‐*Cre*;*Rspo3* ^inv^ female mice and (B) MMTV‐*Wnt1*;*Rspo3* ^inv^ female mice. Left panels: 4× objective; right panels: 20× objective. (C) Number of lung metastases observed per mouse in MMTV‐*Cre*;*Rspo3* ^inv^ (*n* = 21) or MMTV‐*Wnt1* (*n* = 10) cohorts. (D) Representative example of H&E‐stained lung metastasis in an MMTV‐*Cre*;*Rspo3* ^inv^ mouse (20× objective).

To further reveal the features of RSPO3‐driven mammary tumors, we performed immunohistochemical analyses. First, RSPO3‐driven mammary tumors were largely negative for the steroid hormone receptors ERα and PR (supplementary material, Figure S2). We next analyzed expression of cytokeratin‐8 (K8) and cytokeratin‐5 (K5), indicating the luminal and basal compartments, respectively. In RSPO3‐driven mammary tumors, K8 expression was observed throughout solid epithelial tumor structures but in a weak and patchy staining pattern (Figure 3A, upper panel). Also, individual K8‐positive spindle‐shaped cells were observed in EMT regions. K5 expression was found most abundantly in EMT areas and to a lesser extent in solid tumor structures (Figure 3A, lower panel). Thus, the solid epithelial tumor structures harbored weak K8 expression, whereas EMT regions showed K5 expression predominantly. Although RSPO3‐driven mammary tumors contain both basal and luminal keratins, the relatively weak and disorganized expression patterns indicated poor differentiation.

To put this poorly differentiated RSPO3 tumor phenotype into further perspective, we comparatively analyzed WNT1‐driven mammary tumors that developed in the co‐bred MMTV‐*Wnt1*;*Rspo3* ^inv^ cohort (only *Wnt1* transgene expression, given the lack of Cre). WNT1‐driven mouse mammary tumors showed consistent and strong staining for both K8 and K5 in a bi‐layered fashion, clearly segregating luminal and basal cell layers and indicating a distinctive degree of differentiation (Figure 3B). This further emphasized the relatively poor differentiation of mammary tumors driven by RSPO3, together with the typical presence of EMT areas suggesting increased dissemination potential. Therefore, we examined the lungs of mice bearing WNT1‐ or RSPO3‐driven mammary tumors to determine distant metastasis potential. In line with histological features, lung metastases were found in 6 of 21 (29%) mice bearing RSPO3‐driven mammary tumors, mostly presenting in multitude, with up to 25 metastatic lesions per mouse (Figure 3C,D). In contrast, no lung metastases were found in mice with WNT1‐driven mammary tumors (Figure 3C). These findings demonstrate that RSPO3‐driven mammary tumors are poorly differentiated and metastatic.

### RSPO3‐driven mammary tumors are molecularly distinct from WNT1‐driven tumors

**Figure 4:**
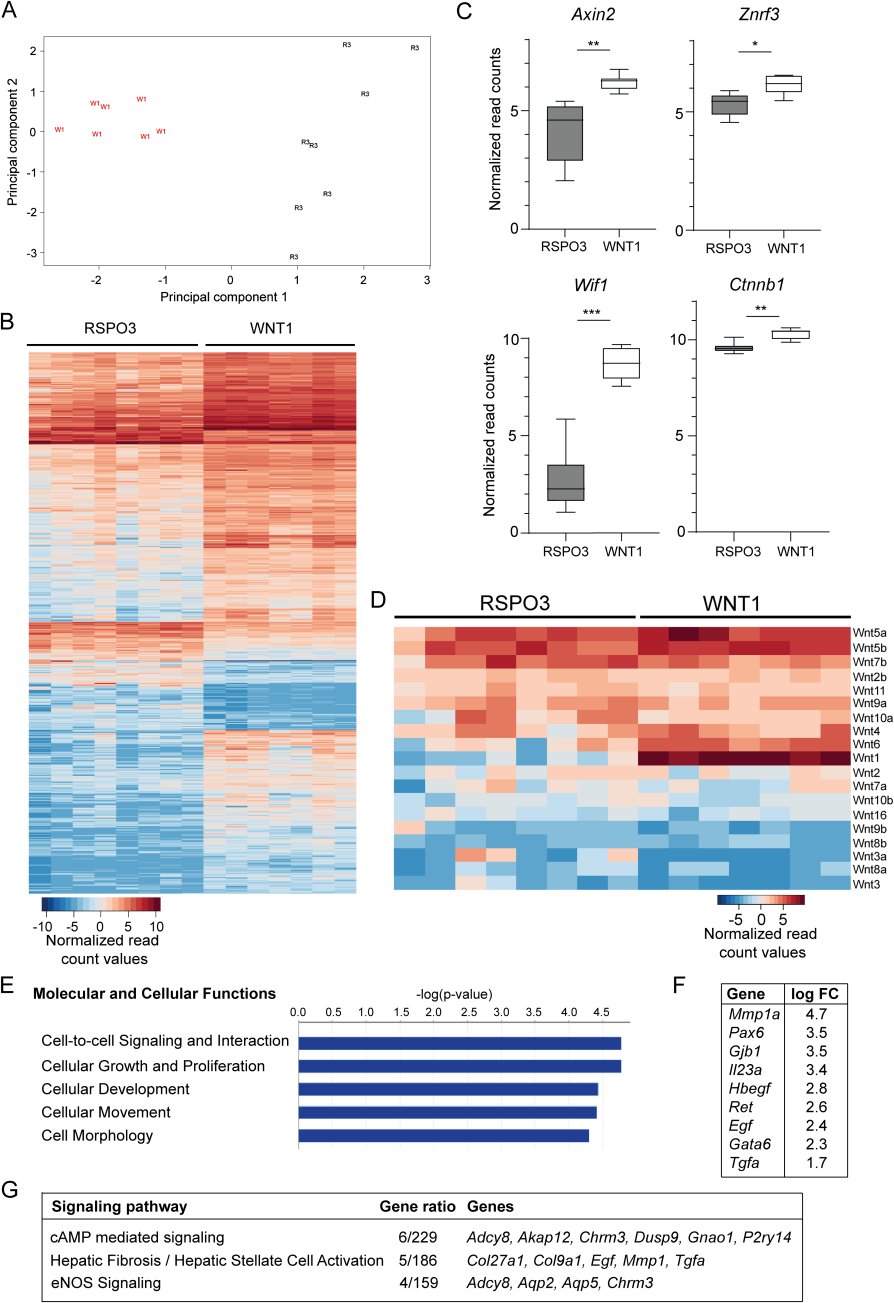
Gene expression analysis of RSPO3‐driven (*n* = 8) versus WNT1‐driven (*n* = 7) mouse mammary tumors. (A) Principal component analysis indicating separate clustering of RSPO3 (R3) tumors from WNT1 (W1) tumors. (B) Heat map illustrating normalized expression values of differentially expressed genes per sample (filtered *p* < 0.05 and log_2_ fold‐change > 1.5). (C) Normalized expression counts of Wnt/β‐catenin pathway genes in RSPO3‐driven (grey boxes) versus WNT1‐driven (white boxes) mammary tumors. Box plots show minimal, median, and maximal expression values and Benjamini–Hochberg adjusted *P* values. *p < 0.05, **p < 0.01, ***p < 0.001. (D) Heat map of normalized expression values of Wnt ligands. (E–G) Gene ontology analysis showing the most significantly enhanced molecular and cellular functions (E), associated upregulated genes and log_2_ fold‐change values (F), and the top three upregulated pathways in RSPO3‐driven mammary tumors (G).

The phenotypic difference between WNT1‐ and RSPO3‐driven tumors might seem striking, since Wnt1 is a classical canonical Wnt ligand driving Wnt/β‐catenin signaling and R‐spondins (RSPOs) are well known to potentiate this same Wnt/β‐catenin route. To look into this further, we assessed the gene expression profiles of RSPO3‐ versus WNT1‐driven mouse mammary tumors by RNA sequencing analysis of the respective mammary tumor tissues. Principal component analysis indicated separate clustering of RSPO3‐ and WNT1‐driven tumors, in line with their distinctive morphology (Figure 4A). Gene expression analysis revealed that 881 genes were differentially expressed, of which 683 genes showed relative upregulation in WNT1 tumors, compared with 198 genes being enhanced in RSPO3‐driven tumors (Figure 4B, filtered *p* < 0.05 and log fold‐change > 1.5). Among these and in line with the above findings, the steroid hormone receptors *Pgr* and *Esr1* were reduced in RSPO3 mammary tumors compared with WNT1 tumors (supplementary material, Figure S3A). With regard to activation of the canonical Wnt pathway, we observed that RSPO3‐driven breast tumors expressed the Wnt/β‐catenin target genes *Axin2*, *Wif1*, *Znrf3*, and *Ctnnb1* itself, however at significantly lower levels than their WNT1‐driven counterparts (Figure 4C). As RSPOs need Wnt ligands to potentiate the Wnt/β‐catenin pathway, we inventoried Wnt ligand expression in the tumors and noticed the presence of a variety of Wnt ligands in both WNT1‐ and RSPO3‐driven tumors (Figure 4D). Compared with RSPO3‐driven tumors, WNT1‐driven tumors showed higher expression of *Wnt1* itself, but also of *Wnt6* and *Wnt5b*. Wnt ligands that were expressed in both tumor cohorts included *Wnt5a*, *Wnt5b*, and *Wnt7b* foremost, in accordance with the reported expression of these ligands in mammary epithelium [18]. Moreover, *Wnt4* was also expressed, which is a crucial cooperator of RSPO1 in Wnt/β‐catenin activation and stem cell expansion in the mouse mammary gland [18, 19]. This indicates that in RSPO3‐driven tumors, endogenous Wnt ligands are available for possible cooperation with RSPO3. Broad expression of Wnt and RSPO receptors was also confirmed (supplementary material, Figure S3B,C).

To obtain a comprehensive insight into the molecular routes that are differentially activated in RSPO3‐driven compared with WNT1‐driven mammary tumors, we performed gene ontology analysis. In RSPO3‐driven tumors, the most significantly enriched molecular and cellular functions were related to cellular signaling, growth, development, movement, and morphology (Figure 4E). The genes that underlie the high ranking of these functions were *Mmp1a*, *Pax6*, *Gjb1*, *Gata6*, and *Ret*, and signaling molecules *Egf*, *Hbegf*, *Il23a*, and *Tgfa* (Figure 4F). The signaling pathways most upregulated in RSPO3‐driven tumors were cAMP‐mediated signaling, hepatic fibrosis/stellate cell activation, and eNOS signaling, involving the upregulation of relatively small sets of genes (Figure 4G). In WNT1‐driven tumors, the molecular and cellular functions that were most significantly upregulated were related to cellular morphology, assembly, signaling, death, and survival (supplementary material, Figure S4A), whereas the top pathways activated in WNT1 tumors were axonal guidance signaling, regulation of the epithelial–mesenchymal transition in development, and human embryonic stem cell pluripotency (supplementary material, Figure S4B). Altogether, in line with the different tumor morphologies of RSPO3‐ and WNT1‐driven mammary tumors, gene expression analysis revealed that their molecular profiles are also distinct.

### RSPO3 and WNT1 co‐expression drives mixed‐phenotype mammary tumors

**Figure 5:**
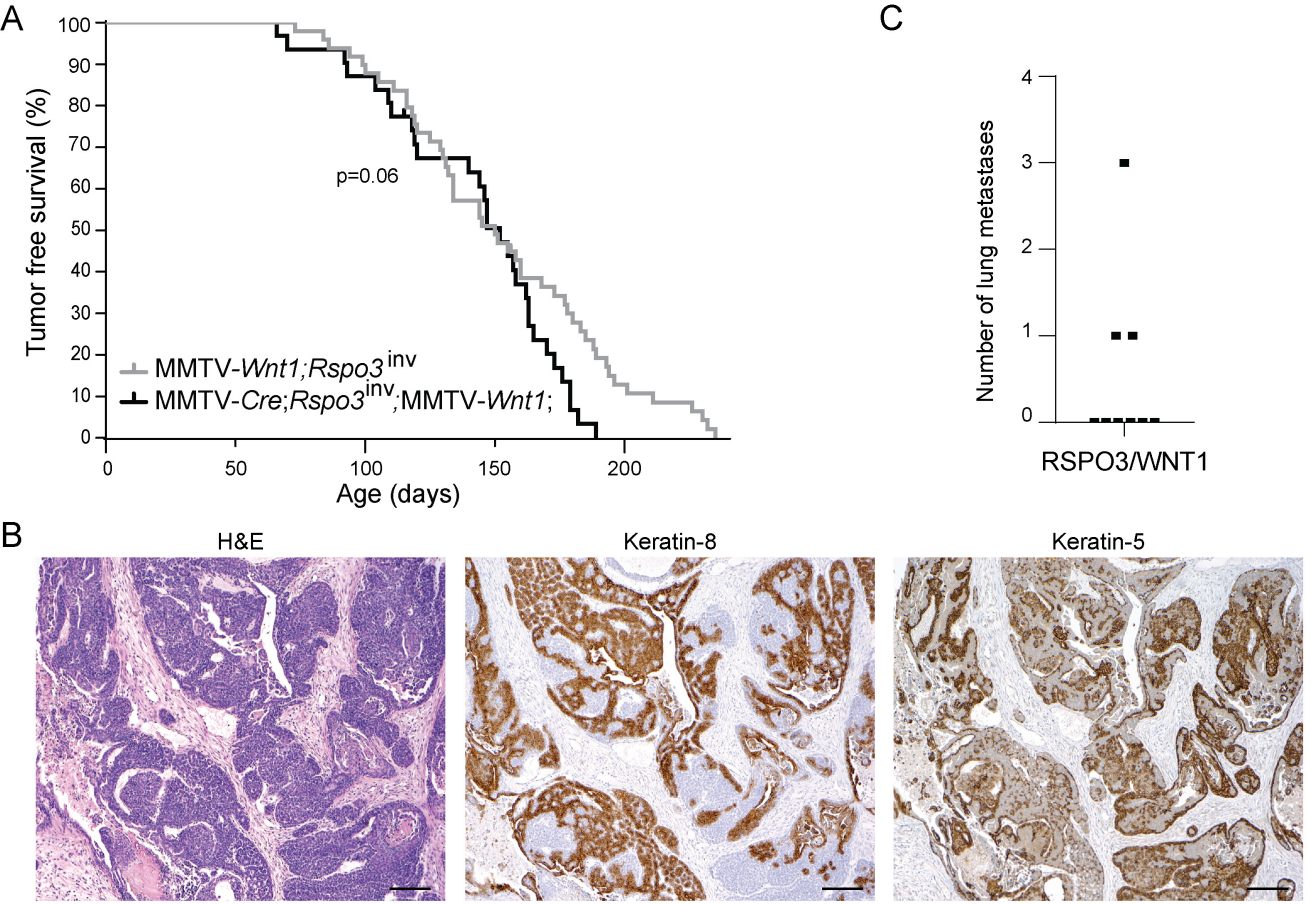
Combined transgenic *Rspo3* and *Wnt1* expression in the mammary gland. (A) Tumor‐free survival curves of mice with expression of the *Wnt1* transgene exclusively (MMTV‐*Wnt1*;*Rspo*3^inv^, *n* = 49) versus both *Wnt1* and *Rspo3* transgenes (MMTV‐*Cre*;*Rspo3* ^inv^;MMTV‐*Wnt1*, *n* = 31) (log‐rank test). (B) Representative RSPO3/WNT1 mammary tumor subjected to H&E, keratin‐8, and keratin‐5 staining (10× objective). (C) Number of lung metastases observed per MMTV‐*Cre*;*Rspo3* ^inv^;MMTV‐*Wnt1* mouse (*n* = 9).

To investigate possible synergism between RSPO3 and WNT1 in the context of mammary tumorigenesis, we generated a cohort of compound MMTV‐*Cre*;*Rspo3* ^inv^;MMTV‐*Wnt1* female mice that expressed both transgenic *Rspo3* and *Wnt1* in their mammary glands (*n* = 31). Compared with *Rspo3* ^inv^;MMTV‐*Wnt1* mice that overexpress the *Wnt1* transgene only (*n* = 49), there was no significant difference in tumor‐free survival (*p* = 0.06), despite a slight trend towards reduced survival (Figure 5A). Histological analysis revealed that mammary tumors developing in mice with RSPO3/WNT1 co‐expression showed a mixed phenotype, typically exhibiting characteristics of both RSPO3‐ and WNT1‐driven tumors (Figure 5B). Grossly, the RSPO3/WNT1 mammary tumors showed a combination of compact solid areas as well as more dilated cystic areas typically seen in RSPO3‐ or WNT1‐driven mammary tumors, respectively (Figure 5B, left panel). In these RSPO3/WNT1 co‐expressing tumors, both keratin‐8 and keratin‐5 were expressed broadly; however, the staining pattern was less organized compared with the bi‐layered staining pattern in WNT1‐driven tumors, indicating reduced epithelial organization and differentiation (Figures 5B and 3C). Thus, RSPO3 co‐expression with WNT1 affects tumor morphology, and accordingly, distant lung metastases were found in three of nine RSPO3/WNT1 mice (Figure 5C). Since no lung metastases were observed in mice with WNT1 overexpression only (Figure 3D), these findings indicate that RSPO3 contributes to WNT1‐driven tumorigenesis by promoting malignant progression.

## Discussion

RSPOs have gained attention as clinically relevant oncogenes, a novel character of RSPOs that has been established especially in the intestinal tract [4]. Considering RSPOs in breast cancer, data indicative of a pro‐tumorigenic role have been reported, though these data remained rather associative and indirect. Among these, *Rspo2* and *Rspo3* have been proposed as potential mammary oncogenes, given their frequent activation in MMTV insertional mutagenesis screens in mice [32, 33, 34, 35]. In breast cancer patients, *RSPO2*, *RSPO3*, and *RSPO4* overexpression has been reported, which is associated with hormone receptor‐negative tumor status and for *RSPO2* also with reduced patient survival [30, 31]. Adding to this, we found that a quarter of breast cancer patients harbor *RSPO2* or *RSPO3* copy number amplification, which is associated with high tumor grade, ER and PR negative status, and reduced survival, indicating the clinical relevance of RSPO. We also provide direct *in vivo* evidence that *Rspo3* acts as an oncogenic driver in the mammary gland, as *Rspo3* overexpression consistently caused the development of mammary tumors in mice. The RSPO3‐driven mammary tumors typically appear as poorly differentiated adenocarcinomas with metastatic potential. These findings establish the oncogenic role of RSPO overactivation in the mammary gland, thus extending the clinical relevance of RSPOs among cancer types.

In colon cancer, *RSPO2* and *RSPO3* gene fusions have been proposed to potentiate Wnt/β‐catenin signaling, providing a mutational alternative for classical *APC* and *CTNNB1* mutations [7]. In our previous study, we showed that *Rspo3* overexpression causes tumorigenesis in the mouse intestine, accompanied by a modest increase in Wnt signaling [5]. Wnt pathway activation has been implicated in tumorigenesis in the breast too, although the underlying mutational causes remain incompletely understood [4, 23]. Conditional *Wnt1* overexpression in the mouse mammary gland is well known to induce mammary tumorigenesis [22]. Since RSPOs are most often envisioned as agonists of the canonical Wnt pathway, we studied our RSPO3 breast cancer mouse model in parallel to the WNT1‐driven counterpart. Strikingly, we found that RSPO3‐driven mammary tumors appeared as completely different entities from those driven by WNT1. Whereas WNT1 was able to drive tumorigenesis faster, RSPO3 tumors were more malignant, showing poor differentiation, areas of EMT, and distant metastases. These morphological differences were further substantiated upon RNA sequencing analysis, which revealed that RSPO3‐ and WNT1‐driven mammary tumors have distinctive molecular profiles. Generally, many more upregulated genes were observed in WNT1‐driven tumors (683) than in RSPO3‐driven tumors (198). Although Wnt/β‐catenin target genes were expressed in RSPO3‐driven tumors, levels were lower than those in WNT1‐driven tumors. Because RSPOs need Wnt ligands to potentiate Wnt/β‐catenin signaling, we examined the presence of endogenous Wnt ligands. We confirmed the expression of several Wnt ligands, including Wnt4, implying that Wnt ligands were available for possible synergy with RSPO3. Despite this, the relatively low expression of Wnt/β‐catenin target genes in RSPO3‐driven mammary tumors suggests that tumorigenesis driven by RSPO3 might be less reliant on Wnt/β‐catenin pathway activation. Instead, or in parallel, RSPO3 might rely on alternative molecular routes and, supportively, 198 genes were upregulated in RSPO3‐driven mammary tumors. At the cellular level, we previously noticed that in the intestine, RSPO3‐driven tumorigenesis was accompanied by a striking expansion of stem cell and niche compartments [5]. Additional studies likewise reported that in the intestine, RSPO3 activation is accompanied by tumorigenic growth and a proliferative stem cell phenotype [6, 37]. This is in accordance with the reported ability of RSPOs to fuel self‐renewal and expansion of stem cells in the intestine [3]. Thus, considering a possible mechanism through which RSPOs contribute to tumorigenesis, most current knowledge is obtained from studies in the intestine and point towards RSPO‐mediated deregulation of the proliferative stem cell compartment. Although the mammary gland differs greatly from the intestine, RSPO is also known to play an essential role in stem cell regulation in this tissue [18, 19]. In the normal mouse mammary gland, RSPO1 has emerged as a key regulator of stem cells, acting with Wnt4 to regulate the expansion of mammary progenitor cells [18, 19]. Comparable to the intestine, RSPO3 overexpression might fuel tumorigenic growth through abnormal expansion of mammary progenitor cells. More research is required to further delineate the molecular and cellular activities through which RSPO3 fuels mammary tumorigenesis.

With this study, we provide *in vivo* evidence for the causal oncogenic capacity of RSPO3 in the breast, extending its clinical relevance beyond the intestine. RSPO3‐driven mouse mammary tumors are distinct from WNT1‐driven counterparts and uniquely present with poor differentiation, malignant transformation, and metastatic potential. Moreover, we found that a quarter of breast cancer patients harbor *RSPO2/RSPO3* copy number amplification, which is associated with worse prognosis and lack of steroid hormone receptor expression, restricting therapeutic options. Targeting RSPO might create a novel window of opportunity for alternative therapeutic intervention in steroid hormone receptor‐negative breast cancer patients and thereby provide significant clinical benefit. As therapeutic anti‐RSPO antibodies already exist and anti‐RSPO3 has been demonstrated to be well tolerated in a clinical trial for colon cancer, realistic and relatively fast opportunities lie ahead to explore RSPO targeting in breast cancer patients.

## Author contributions statement

ERMB and JH conceived, designed, and supervised the project. MB, NCT, CMEA, ERMB and JH carried out mouse studies. J‐YS, EJtS, ERMB, JH and PWBD were responsible for histology. PWBD interpreted results and provided input. ERMB, EJtS and JH analyzed gene expression. EJtS and ERMB carried out the *in silico* analysis. ERMB and EJtS wrote the manuscript.

## Supporting information


**Figure S1.** Regulation of *Rspo3* transgene expression in the *Rspo3* ^inv^ mouse model
**Figure S2.** Steroid hormone receptor staining in an RSPO3‐driven mouse mammary tumor
**Figure S3.** RNA expression analysis of receptors for steroid hormones, Wnt, and RSPO in RSPO3‐ and WNT1‐driven mammary tumors
**Figure S4.** Gene ontology analysis showing the most significantly enhanced molecular and cellular functions and the top three upregulated pathways in WNT1‐driven mammary tumors

## Data Availability

The data that support the findings of this study are available from the corresponding author upon reasonable request.
