# Supplementary material for: R‐spondin‐3 is an oncogenic driver of poorly differentiated invasive breast cancer
Source: J Pathol. 2022 Sep 15;258(3):289–99. doi: 10.1002/path.5999 (PMC9825844; doi:10.1002/path.5999)
Supplement: Supplementary file 1 — Figure S1. Regulation of Rspo3 transgene expression in the Rspo3 inv mouse model Figure S2. Steroid hormone receptor staining in an RSPO3‐driven mouse mammary tumor Figure S3. RNA expression analysis of receptors for steroid hormones, Wnt, and RSPO in RSPO3‐ and WNT1‐driven mammary tumors Figure S4. Gene ontology analysis showing the most significantly enhanced molecular and cellular functions and the top three upregulated pathways in WNT1‐driven mammary tumors [file PATH-258-289-s001.docx]

**R-spondin-3 is an oncogenic driver of poorly differentiated invasive breast cancer**

EJ ter Steege *et al.* *J Pathol*, <https://doi.org/10.1002/path.5999>


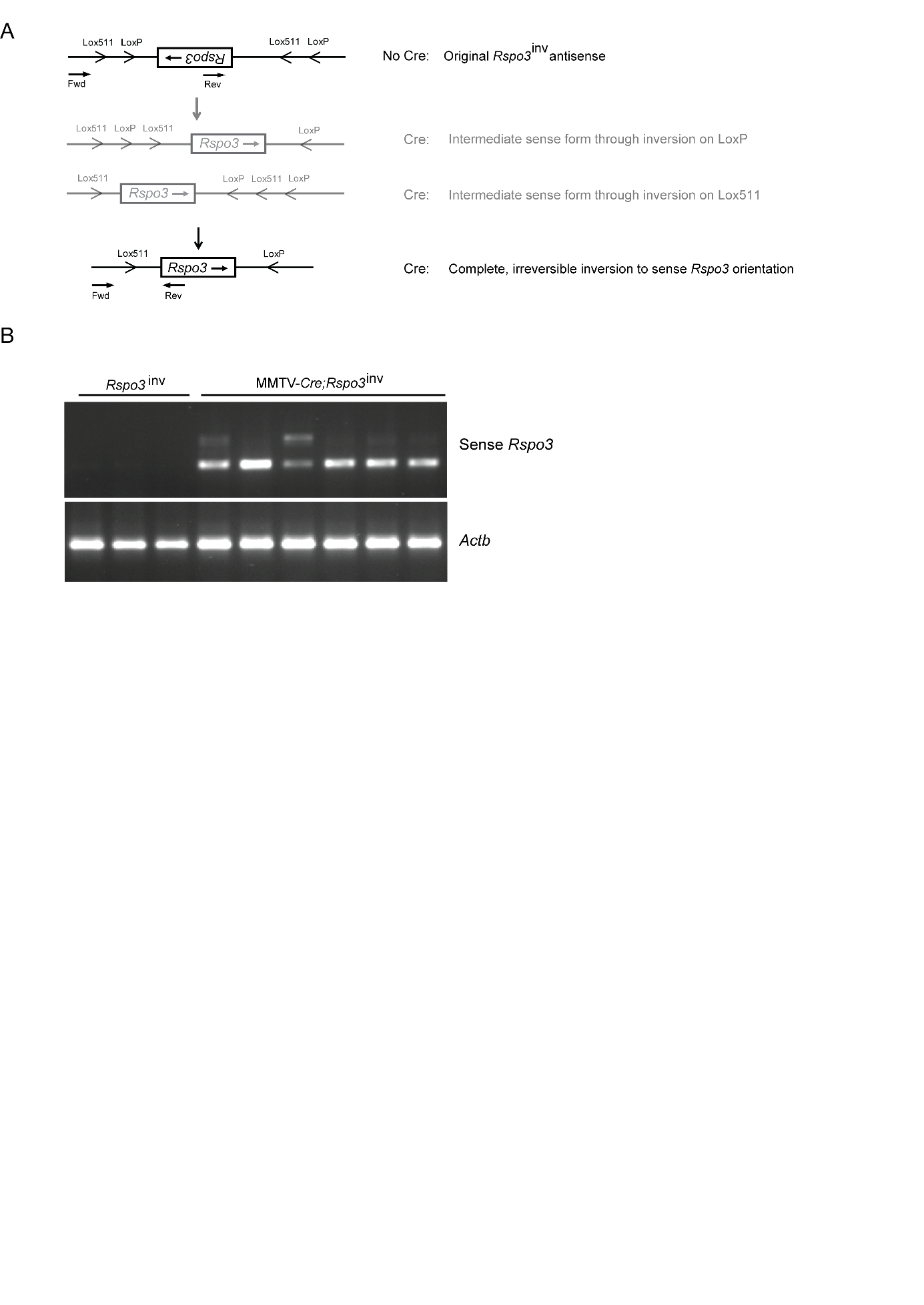


**Figure S1.** Regulation of *Rspo3* transgene expression in the *Rspo3*^inv^ mouse model. (A) Simplified scheme of the original *Rspo3*^inv^ construct and resulting derivatives upon Cre activity. Cre mediates inversion of the *Rspo3* coding sequence using either the *LoxP* or *Lox511* sequences that are oppositely oriented, giving intermediate forms with sense *Rspo3* orientation that can revert back to antisense. Further Cre activity causes excision of the remaining sequences between homologous *Lox* sites that are now oriented in equivalent direction. This provides the final product with irreversible sense orientation of the *Rspo3* transgene. Figure adapted from ref 5 with permission of BMJ Publishing Group Ltd. Fwd and Rev arrows indicate the location and orientation of the primers used to detect mRNA expression of the *Rspo3* transgene. (B) RT-PCR confirming sense-oriented *Rspo3* mRNA expression in mammary gland tissues of MMTV-*Cre*;*Rspo3*^inv^ but not *Rspo3*^inv^ mice. Upper bands represent the intermediate products and lower bands the final, locked product.


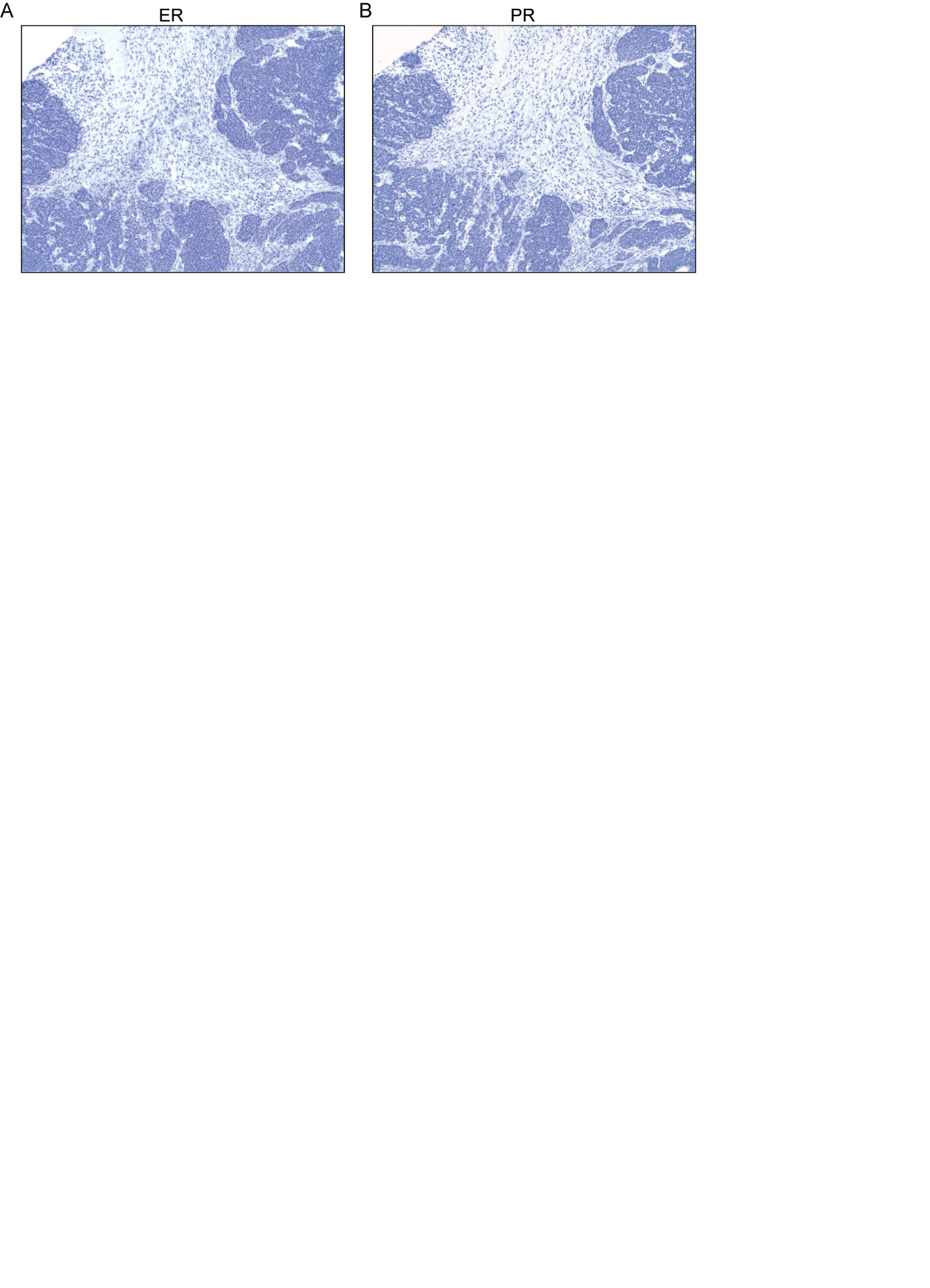


**Figure S2.** Steroid hormone receptor staining in an RSPO3-driven mouse mammary tumor. Representative immunohistochemical staining of an RSPO3-driven mouse mammary tumor for (A) ERα and (B) PR.


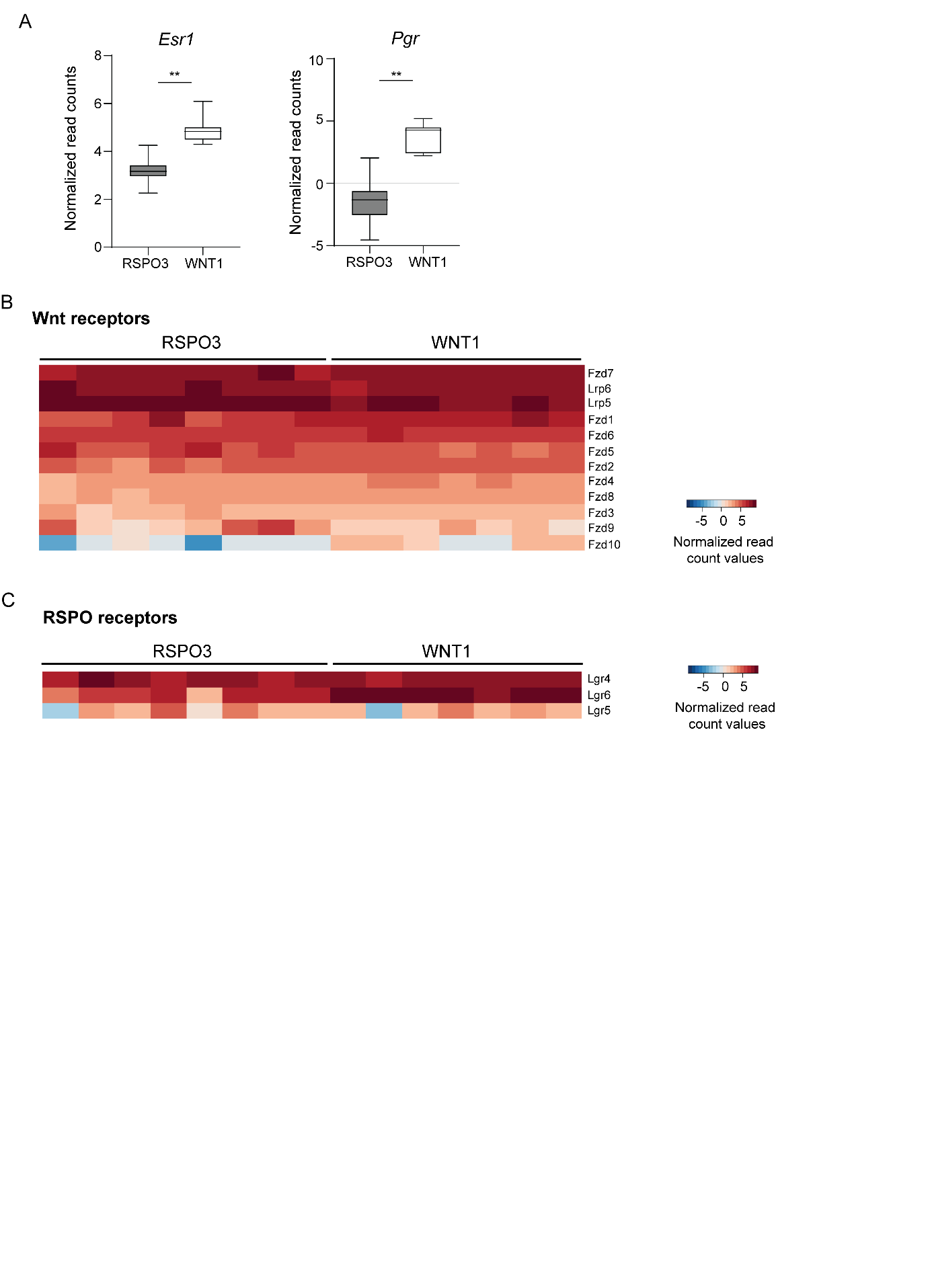


**Figure S3.** RNA expression analysis of receptors for steroid hormones, Wnt, and RSPO in RSPO3- and WNT1-driven mammary tumors. (A) Normalized expression counts of estrogen- and progesterone receptors in RSPO3-driven (grey boxes) versus WNT1-driven (white boxes) mammary tumors. Box plots show minimal, median, and maximal values, and adjusted *P* values. ***p* < 0.01 (Benjamini–Hochberg method in R). Heat maps illustrating normalized expression values of (B) Wnt and (C) RSPO receptors.


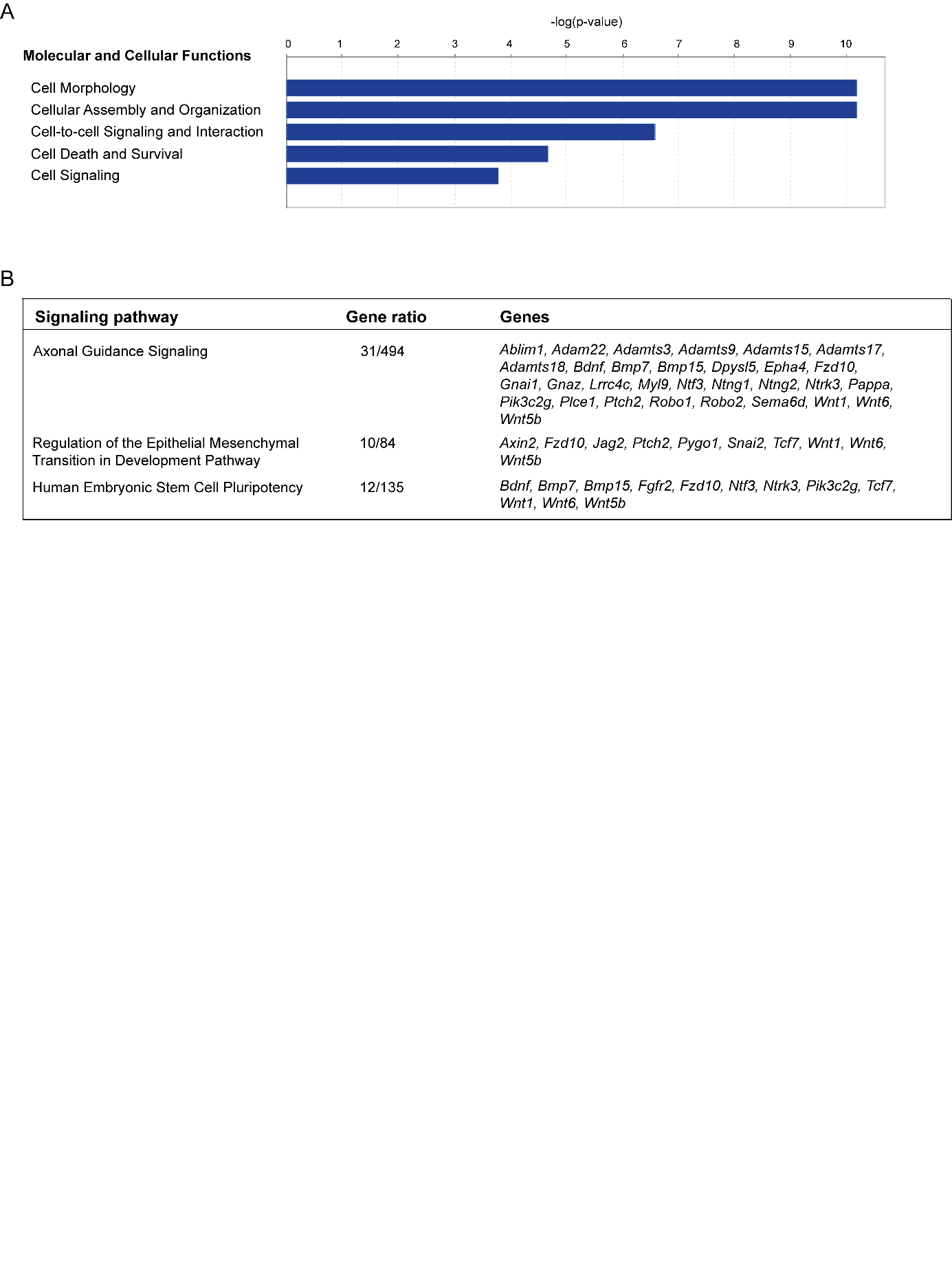


**Figure S4.** Gene ontology analysis showing the most significantly enhanced molecular and cellular functions (A) and the top three upregulated pathways (B) in WNT1-driven mammary tumors.
